# Airway remodeling in chronic obstructive pulmonary disease: characteristics and opportunities

**DOI:** 10.3389/fmed.2025.1556868

**Published:** 2025-07-28

**Authors:** Minjie Pan, Xiaojing Zhou

**Affiliations:** ^1^Department of Respiratory and Critical Care Medicine, Zhoushan Hospital, Zhoushan, China; ^2^Department of Neurology, Zhoushan Hospital, Zhoushan, China

**Keywords:** COPD, airway remodeling, epithelial-mesenchymal transition, inflammation, oxidative stress

## Abstract

Chronic obstructive pulmonary disease (COPD) is a chronic respiratory disease characterized by irreversible airway remodeling and is a global burden on the healthcare system. The World Health Organization predicts it will be the third leading cause of death by 2030. The causes of airway remodeling in COPD are complex. Several elements, such as the lung parenchyma and interstitium, as well as endothelium, mesenchymal cells, and a range of bioactive chemicals, work together to either encourage or impede the alteration of the airway’s structure during the remodeling process. Airway remodeling is an important factor in the irreversible limitation of ventilatory function. To reduce airway remodeling, significant efforts are being directed to find effective therapeutic ways that inhibit airway remodeling. In China, many patients use traditional Chinese medicine (TCM). Some TCM can improve the symptoms and lung function of COPD patients. Here, we describe the molecular mechanisms and key cellular players of airway remodeling in COPD patients and review the Chinese herbal medicines that may effectively inhibit airway remodeling.

## 1 Introduction

The global recognition of COPD is growing due to its widespread nature, and it poses a risk to one’s life and well-being. A study shows the prevalence of COPD in the United States from 2007 to 2012 was 13.1–14.3% (1). Also, in China, COPD causes a significant financial and medical burden. COPD is typified by an irreversible reduction in lung function and is linked to particulate matter inhalation, as well as genetic, developmental, and social variables (2, 3). There are various mechanisms involved in the pathophysiologic alterations in COPD, such as the development of emphysema and minor airway lesions, which cause an irreversible loss in lung function. The hallmark of COPD is airway remodeling. Under normal physiological conditions, tissue remodeling also occurs, mediating a mild response to inflammation while fostering tissue growth and mending damage. On the other hand, excessive remodeling in pathological situations may result in modifications to pertinent structures, which could impact organ function and complicate follow-up treatment. Structural alterations define airway remodeling and result from dysregulated tissue formation and breakdown. Causes of structural changes include disorders of angiogenic factors, deposition and fibrosis of subepithelial matrix proteins, proliferation and hypertrophy of smooth muscle cells in the airways, epithelial abnormalities, epithelial–mesenchymal transition (EMT), which result in thickening of the airway wall and airflow restriction (4–6). Various cytokines, chemokines, growth factors, and other bioactive substances participate in this process (6–8). Fibrosis in COPD occurs mainly in the airway wall and predominantly in the small airways (6, 9). To better understand airway remodeling in patients with COPD, this article briefly reviews the pathological mechanism of airway remodeling, describing the relationship between oxidative stress and multiple pathophysiologic changes during airway remodeling. TCM has a long history in China and plays a role in the treatment of COPD. In this review, we discuss several important components to highlight the role of TCM in airway remodeling.

## 2 Definition and evaluation of airway remodeling

Airway remodeling refers to both quantitative and qualitative modifications of the airway wall. Epithelial damage, goblet cell hyperplasia, enlargement of submucosal mucous glands, and mucus hypersecretion can be observed in the airway lumen. Fibroblast activation and proliferation, extracellular matrix (ECM) modifications, angiogenesis and vascular remodeling, and airway smooth muscle (ASM) proliferation and hypertrophy are among the alterations in the subepithelial structure of the airways (9, 10). Airway remodeling can be assessed by non-invasive tools, including high-resolution computed tomography (HRCT), positron emission tomography (PET) scan, single-photon emission computed tomography (SPECT), optical imaging with fluorescence and bioluminescence (11). Several indicators like airway wall area percentage, lumen diameter, lumen thickness, and airway lumen area, can help to evaluate airway remodeling (12–14). Airway MRI has low resolution, however, by inhalation of 129*X_e_*, airway remodeling can also be sensitively measured (15, 16). Optical coherence tomography (OCT) is a non-invasive, high-resolution optical imaging technique. When OCT and bronchoscopy are used together, the airway wall can be seen in detail, and peripheral airway remodeling can be positively identified (17–19).

In recent years, an overlap phenomenon has been observed in asthma and COPD, where symptoms are similar to those of COPD but respond significantly to bronchodilators, called asthma-COPD overlap (ACO). A 10–40% of patients with COPD and 15–35% of patients with asthma may have an ACO (20). A study using low-dose CT analysis to assess proximal airway remodeling, emphysema, and air trapping in patients with ACO found that lung function indices (FEV1, FEV1/FVC, etc.) were significantly lower in patients with ACO than in patients with non-severe asthma, suggesting that the degree of airflow obstruction was more severe in patients with ACO. The degree of emphysema was more severe in patients with ACO than that in patients with non-severe asthma, while was similar to that in patients with mild to moderate COPD (21–23). Using multi-row spiral CT (MDCT) to assess airway wall thickness and airway lumen area in patients with ACO and asthma, researchers found that airway wall thickness was significantly greater in the ACO group compared to the asthma group and healthy controls. Overall, the degree of lung function impairment and airway remodeling in patients with ACO was intermediate between those with asthma and COPD (24).

## 3 Physiological factors affecting airway remodeling

Chronic obstructive pulmonary disease patients are predominantly in the elderly population. The prevalence of COPD increases with age, and senescence is an independent risk factor for COPD. Structural cells such as airway epithelial cells, ASM cells, and fibroblasts undergo senescence with age, which is characterized by decreased cell proliferation, impaired function, and secretion of senescence-associated secretory factors (SASPs). SASPs can contribute to inflammatory responses, ECM deposition, and proliferation of ASM. Aging also leads to decreased antioxidant capacity and increased oxidative stress, immune dysfunction, and increased inflammatory response, which leads to thickening and reduced elasticity of the airway wall (25). Aging and airway remodeling reinforce each other in a vicious cycle.

Usually, the human respiratory tract is inhabited by a complex mixture of microorganisms. The composition of the lung microbiota is dynamic and plays a crucial role in regulating innate and adaptive lung immunity. It has been found that in normal lung tissue, common bacteria include Prevotella spp, Streptococcus spp, Veronica spp, Aspergillus spp, Proteus spp, Neisseria spp, Clostridium spp, Haemophilus influenzae spp, Rochesteria spp, Actinomyces spp, Streptococcus pepticus spp, Corynebacterium spp, and Pseudomonas spp. Common fungi include Candida spp, Saccharomyces cerevisiae, Penicillium spp, and Dendrobatrachium spp. And viruses such as Cycloviridae, Iridoviridae, Herpesviridae, and Poxviridae are found (26). Compared to other parts of the body, like the gut, the microbial density of normal lung tissue is low, and its composition changes dynamically with time, the environment, and the host’s health status. In patients with COPD, dysbiosis of the lung flora can lead to colonization by pathogens such as Staphylococcus aureus and Gram-negative bacteria. These pathogens can trigger an inflammatory response, leading to the production of harmful metabolites such as lipopolysaccharides (LPS) and short-chain fatty acids (SCFAs), resulting in overactivation of pro-inflammatory Th17 cells and dysfunction of regulatory T-cells (Tregs), leading to lung tissue damage and dysfunction. Lung dysbiosis can also affect the function of immune cells, such as neutrophils and macrophages, as well as the levels of immunomodulatory factors, resulting in a dysregulated immune response that promotes inflammation and tissue damage (27).

## 4 Oxidative stress promotes airway remodeling

In COPD, Oxidative stress has been found to have a crucial role in the development of airway remodeling (28–30). Mitochondrial dysfunction leads to decreased oxidative phosphorylation, increased Reactive Oxygen Species (ROS) production, and changes in cell metabolism, which are increasingly recognized as essential events in the pathogenesis of a series of lung diseases, including COPD (31, 32).

Mitochondria are the center of oxidative stress in cells and the major ROS source. Mitophagy is a process by which cells selectively degrade damaged mitochondria through an autophagy mechanism, to maintain mitochondrial and intracellular homeostasis (33). The PINK1/Parkin signaling pathway predominantly regulates mitochondrial autophagy. Under physiological conditions, PINK1 is translocated to the outer mitochondrial membrane, where it remains stable. However, when mitochondrial damage occurs, the loss of membrane potential results in the accumulation of PINK1 on the surface of the mitochondria. This accumulation facilitates the recruitment of the Parkin protein, which tags the damaged mitochondria for degradation. Consequently, this process initiates the formation of autophagosomes that ultimately lead to the lysosomal degradation of the impaired mitochondria (34). Mitophagy plays a dual role in the development of lung diseases (35). In a physiological state, mitophagy keeps the population of mitochondria and is crucial in protecting cells against disease (36). Mitophagy has been suggested to play a protective role in the development of COPD in some studies (37–39). The pathological state of mitophagy aggravates the inflammatory reaction. After exposure to cigarette-smoke (CS), mitochondrial autophagy in airway epithelial cells is increased to clear damaged mitochondria, and excessive autophagy contributes to the progression of COPD (40). Mitochondrial protein Nix is a selective autophagy receptor. When cigarette extract stimulated airway epithelial cells, the expression of Nix protein increased. Nix overexpression can enhance mitophagy and aggravate the damage to mitochondria and cells (41). Mitophagy can also promote pulmonary fibrosis, which is related to airway remodeling (42). Cigarette-smoke extract (CSE) up-regulates mitophagy in ASM through ERK1/2 phosphorylation. The imbalance of ASM cell mitophagy induced by CS may be one of the mechanisms contributing to airway remodeling in COPD (43, 44). However, some studies have shown that mitochondrial autophagy is impaired in patients with COPD. Studies have demonstrated that the levels of Parkin are diminished in the lung tissue of COPD patients. Furthermore, silencing PINK1 and Parkin in bronchial epithelial cells results in a reduction of mitophagy and an increase in the production of ROS (37).

Reactive Oxygen Species induce lipid peroxidation and produce toxic products such as malondialdehyde, which can damage proteins by causing protein cross-links and stimulate lung inflammation, leading to alveolar wall destruction (45). CS can activate cells via toll-like receptor 4 (TLR4) while altering the mitochondrial activity of airway epithelium and promoting the formation of free ROS (46). Endoplasmic reticulum stress (ERS) refers to the impairment of the normal function of the endoplasmic reticulum (ER), accumulating unfolded or misfolded proteins and disrupting Ca^2+^ homeostasis. In COPD patients, ERS promotes oxidative stress and increases ROS production by inhibiting the expression of antioxidant enzymes, promoting the expression of ROS-generating enzymes, and raising the expression of oxidoreductase cytochrome P450 (47).

Oxidative stress causes increased cell proliferation, including ASM, in COPD patients (28, 48, 49). Hypertrophic hyperplasia of ASM is mainly seen in the peripheral remodeled airway (50, 51). When ASM malfunctions, it releases inflammatory mediators. ASM cells have multiple functions, including contraction, proliferation, and migration, termed phenotypic plasticity. This regulation of phenotypic transitions is accomplished through complex coordinated interactions between external stimuli (52). It has been demonstrated that oxidative stress and peroxide encourage ASM to contract and proliferate, encouraging airway remodeling in COPD (53, 54). In addition, lipopolysaccharide, ECM, and growth factors also contribute to the increased ASM proliferative phenotype (55–57). Potassium-calcium channels are present in ASM cells. *KCa*_3.1_ and *KCa*_1.1_ are two channels with different conductance. *KCa*_3.1_ plays a critical role when activated by calcium ions, maintaining cell viability through the exchange of potassium and calcium ions. Freshly isolated human ASM cells express large conductance *KCa*_1.1_ channels (58). A loss of *KCa*_1.1_ channels and gain in *KCa*_3.1_ channels occurs when ASM cells switch from a contractile to a proliferative phenotype. This was observed in platelet-derived growth factor-induced activation of asthmatic human bronchial smooth muscle cells (58, 59). *KCa*_3.1_ Blocking may at least partially prevent ASM reconfiguration (59).

Hypertrophic and hyperplastic ASM cells exhibit metabolic changes, including increased glutamine metabolism, impaired fatty acid oxidation (FAO), and reduced ATP levels. Glutamine serves as a precursor in the biosynthesis of glutathione. An elevation in glutamine levels may facilitate the production of the glutathione, an important intracellular antioxidant, thereby augmenting the antioxidant capacity of cells and diminishing the concentration of ROS (48).

## 5 Airway inflammation and immune response accelerate airway remodeling

T lymphocytes, macrophages and neutrophils are the primary inflammatory cells that infiltrate the airways of COPD patients (9, 60). The neutrophil is increased in sputum and bronchoalveolar lavage fluid (BALF) from patients with COPD, and its recruitment to the airways and parenchyma requires the initial adhesion of E-selectin to endothelial cells. The expression of E-selectin was up-regulated in airway endothelial cells of COPD patients (61). The neutrophil secretes serine endopeptidase, including neutrophil elastase, cathepsin, and protease, as well as MMP-8 and MMP-9, which may contribute to alveolar destruction (62). T lymphocytes and macrophages predominated in the airway wall of COPD patients, with CD8^+^ T lymphocytes dominating the T lymphocyte phenotype. This is not the same as the asthma patients’ airway CD4^+^ T lymphocyte infiltration (63).

Patients with COPD had increased T lymphocytes in their lung parenchyma, peripheral blood, and central airways. The rise in CD8^+^ T cells was larger than in CD4^+^ T cells (64). Mice lacking B or T cells failed to develop airway remodeling, suggesting the importance of lymphocytes in airway construction (65). Patients with COPD have higher expression levels of CXCR3, a receptor for the chemokines CXCL9/10/11, in their lung T cells. The accumulation of CD4^+^ T and CD8^+^ T cells may be facilitated by increased production of CXCL10 in bronchiolar epithelial cells (66). Patients with COPD also have more lymphocytes in their lungs. B-lymphocytes exist as lymphoid follicles in the lung parenchyma and the surrounding airway (64). Decreased expression of CXCL13, a B-cell chemokine, attenuated CS-induced BALF inflammatory cells and partially protected the alveolar wall from destruction, but did not affect the development of airway remodeling (67).

Macrophages appear to play a key role in COPD. COPD patients show higher levels of macrophages and macrophage chemoattractant in their lung parenchyma and airways (68). Based on surface molecules and distribution, macrophages in the lung can be classified as either interstitial macrophages (IM) or alveolar macrophages (AM) (69). AM is found in the lungs’ air spaces, while IM is found in the spaces between the alveoli and blood vessels. While both AM and IM are macrophages with increased phagocytic capacities, AM is regarded to have a more robust capacity (70, 71). When AM encounters IFN-inducible protein-10 and IFN-γ-induced cytokines, they can release MMP-12, accelerating tissue deterioration and elastic degradation (72). Laminin A1 is strongly expressed on AM and is induced by TGF-β, has been reported to be overexpressed in idiopathic pulmonary fibrosis patients (73). AM from smokers with COPD were classified into M1 and M2 phenotypes. Type 2 immune response, also called adaptive immunity, is mediated by M2. In patients with COPD, macrophages tended to differentiate into M2 cells (74). Pathological remodeling of the pulmonary airway can result from an overabundance of type 2 immune responses (73, 75). The development of COPD is closely associated with the imbalance of M1 and M2 in the airways (69). Peroxisome proliferation-activated receptor-γ (PPAR-γ), a nuclear transcription factor essential to AM polarization, is a member of the nuclear hormone receptor superfamily (76). In COPD patients, there is dysregulation of PPAR-γ expression. In COPD mice, stimulation of PPAR-γ reduces airway remodeling and enhances AM’s polarization stability (77). As a result, maintaining the normal structure of the airways requires controlling the balance between M1 and M2 (Figure 2). In addition to the imbalance of macrophages, the metabolic characteristics of AMs change significantly. CS causes Ams and neutrophils to generate a large amount of ROS, leading to mitochondrial dysfunction and increased oxidative stress. And in COPD patients who smoke, this phenomenon persists even after they quit smoke (78). Abnormal iron metabolism in AMs, including reducing iron-chelating proteins, also leads to iron accumulation and increased ROS production (79).

## 6 Oxidative stress can cause airway remodeling by promoting EMT

An essential part of the airway’s innate immune system is the epithelium. It is the fundamental barrier of the airway. The airway epithelium consists of four cell types: basal cell, ciliated cell, secretory cell, and intermediate cell (80). Several processes help epithelial repair when the healthy airway epithelium is harmed or when an inflammatory response occurs: 1. Migration of epithelial cells to the injury site. This process can be regulated by integrin-mediated activation of transforming growth factor-β (TGF-β) and regulation of the WNT/β-catenin signaling pathway (81, 82). 2. Matrix components are deposited at the site of injury, these include ECM glycoproteins, fibronectin, and vitronectin, as well as basement membrane components such as laminin and collagen IV (81, 83). 3. Mesenchymal stem cells differentiate into epithelial stem cells during the repair process and then rebuild various epithelial cells to ensure the integrity of the airway epithelium (84). The loss of integrity in the airway epithelium results in exacerbated tissue permeability and immunological activation. Airflow blockage and microbiological overgrowth are caused by excessive mucus secretion and poor mucociliary clearance (85).

**FIGURE 1:**
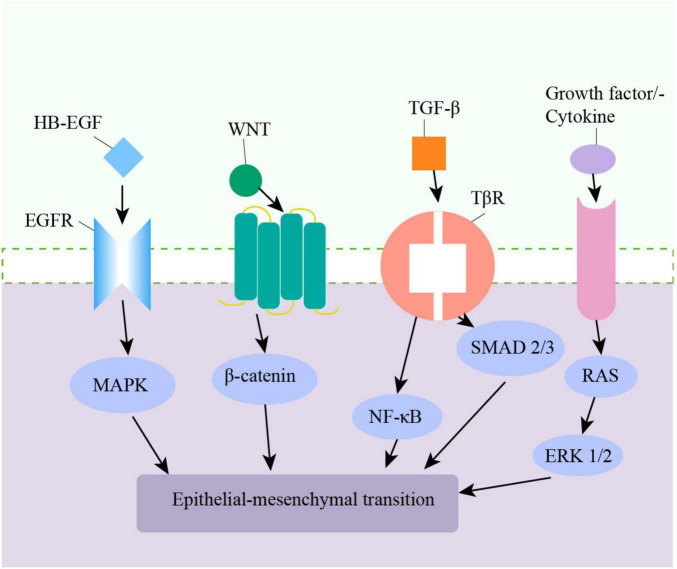
Multiple pathways in the airway lead to epithelial–mesenchymal transition (EMT).

The pathologic process of epithelial cells changing into mesenchymal cells by phenotypic transformation is known as EMT, and it is a commonly acknowledged mechanism for modifying airway walls. During the EMT process, epithelial cells lose polarity, acquire markers of mesenchymal cells, and can migrate (86, 87). EMT is commonly categorized into three types. Type I EMT is associated with embryogenesis and organ development. Type II EMT is linked to tissue repair and chronic inflammation-associated wound healing. Type III EMT is responsible for malignant epithelial cells acquiring a migratory phenotype (88, 89). Type II EMT primarily affects the small airways, mediating small airway fibrosis (89). CS induces EMT in bronchial epithelial cells in multiple ways, including producing TGF-β1, increasing oxidative stress, phosphorylating ERK1/2, and SMAD2/3 (90–92). Oxidative stress plays a key role in the pathogenesis of CS-induced EMT in the airway epithelium by promoting the expression of EMT-related transcription factors and promoting the degradation of extracellular matrix (47). ROS can affect TGF-β1-induced EMT in A549 cells (93, 94). Also, the Wnt/β-Catenin and NF-κB signaling pathways are involved in airway epithelial EMT in COPD patients (95–97) (Figure 1). Monocytes in the lung can stimulate EMT in lung epithelial cells through the Toll-like receptor 2/matrix metalloproteinases -9(TLR2/MMP-9) axis (98). Heparin-binding EGF-like growth factor (HB-EGF) is the ligand of the EGF super-family and is up-regulated in the sputum and lung tissue in COPD patients. It is thought to induce EMT in bronchial epithelial cells (99, 100). Additionally, EMT is believed to represent a novel source of fibroblasts, promoting ECM deposition by increasing fibroblast accumulation (101). Keratin 15 (KRT15) is a type of keratin that is closely related to the repair and regeneration of tissue cells after injury. KRT15 can inhibit EMT by promoting MMP-9 expression and protecting the alveolar structure, and is a potential target for the treatment of COPD (102, 103).

**FIGURE 2:**
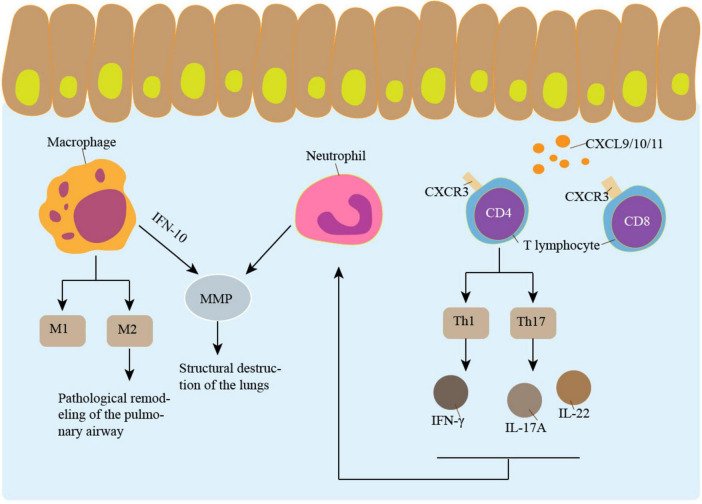
Inflammation promotes airway remodeling. Macrophages, neutrophil and lymphocyte are involved in airway remodeling. M2 type macrophages play an important role in airway remodeling. IL-10-induced MMP secretion by macrophages is involved in the pathogenesis of emphysema. The CXCL9/10/11-CXCR3 Axis recruits CD4 + T cells and CD8 + T cells. CD4 + T cells secrete a variety of cytokines that participate in airway remodeling and promote neutrophil aggregation. Neutrophil increases emphysema through MMP.

### 6.1 Airway remodeling is closely related to ECM abnormality

One of the consequences of EMT is ECM oversecretion. The ECM regulates tissue development and homeostasis (104). ECM shapes the thickness and elasticity of the airway wall. Abnormal buildup of ECM proteins may result in airway remodeling, for example, increased synthesis and breakdown of ECM (105). The most prevalent components in the ECM of COPD patients include proteoglycan (PGs), glycosaminoglycan (GAGs), collagen, and elastin. These components significantly impact lung tissue’s composition and function, and interact when tissue is injured (106, 107).

The lung’s three main ECM compartments are the alveolar interstitium, the foundation membrane, and the airway lamina propria. The main protein components of the basement membrane are collagen IV and laminin. Fibrillar collagen, elastin, fibronectin, glycosaminoglycans, and proteoglycans are the main components found in the lamina propria and interstitium. Fibroblasts and activated myofibroblasts are the primary ECM-producing cells in the lung. Endogenous proteases, of which matrix metalloproteinases (MMPs) are the predominant kind in the lung, control the destruction of ECM (108). It has been demonstrated that MMPs, such as MMP-1, -2, -7, -9, and -12, destroy a range of matrix elements, including collagen and elastin. Elastin is the main target of disintegration in the process of remodeling. The degradation of elastin is a major cause of emphysema, resulting in a loss of lung tissue elasticity. Collagen V and VI are involved in the formation of the reticular basement membrane, which serves to preserve the shape of the lungs, and their increased expression in the airways of COPD patients may result in airway wall thickening and airflow limitation. Furthermore, chondroitin sulfate demonstrated a considerable increase in BALF during COPD exacerbation, which could be linked to airway remodeling (109). Although the precise function of MMP-12 is uncertain, it is significant since in animal models, small airway remodeling can be inhibited by MMP-12 knockdown or MMP-9/-12 inhibitors (110–113). Oxidative stress can cause changes in ECM expression, and higher levels of oxidative stress may promote collagen buildup. In cardiac fibrosis, elevated levels of oxidative stress have been linked to increased amounts of collagen, and antioxidant therapy has been proven to lower collagen levels (114).

### 6.2 Transforming growth factor-β is essential for fibrosis

Transforming growth factor-β is a cytokine known to increase ECM production (115). TGF-β1 is a key component in fibrotic conditions and is necessary for developing myofibroblasts. It is produced by mast cells and granulocytes. Elevated ROS levels activate TGF-β1, which stimulates the fibrotic response (116). ROS can stimulate TGF-β1 secretion from airway epithelial cells, release the TGF-β1 receptor from the latency-associated peptide (LAP) complex by dissociation from LAP or activate matrix metalloproteinases (117, 118) TGF-β1 primarily activates cytosolic Smad protein via transmembrane receptor serine/threonine kinase. The activation of the TGF-β1-induced α-SMA promoter and the *in vitro* expression of α-SMA protein were markedly enhanced by SMAD3 overexpression (119). The expression of α-SMA, a hallmark of mature myofibroblasts, regulates fibroblasts and encourages contraction of the wound edge (120). Myofibroblast apoptosis is reduced in pathological situations, ECM formation persists, and breakdown is impeded, resulting in tissue sclerosis (107). Several studies have indicated that there are more α-SMA positive cells in the bronchi of COPD patients (121, 122). In circulation, the level of α-SMA was also significantly increased in patients with COPD (123). The TGF-β-independent pathway induced by IL-4 and IL-13 can initiate the transformation of resident fibroblasts into myofibroblasts. It has also been demonstrated that IL-4 stimulates the synthesis of collagen, which encourages airway remodeling even more (107).

## 7 Oxidative stress and necroptosis interact to promote airway remodeling

Cigarette-smoke induces numerous mechanisms of cell death, and the pattern of cell death dictates when inflammatory reactions start. Necroptosis involves greater inflammatory reactions and the release of damage-associated molecular patterns (DAMPs) than apoptosis (124). Necroptosis is a form of programd inflammatory cell death (39). It is characterized by organelle enlargement, plasma membrane disruption, and cytolysis (125). The route involved in necroptosis is mainly dependent on receptor-interacting protein kinase (RIPK) and Mixed lineage kinase domain-like protein (MLKL), which are also the pathway’s primary detecting proteins (126). RIPK3, RIPK1, and MLKL create a multiprotein complex called the necrosome (127). RIPK1 recruits and phosphorylates RIPK3 to form ripoptosome, but this is not absolute, and there is evidence of RIPK1-independent necroptosis. Then phosphorylates MLKL to form necrosomes (128).

Oxidative stress and necroptosis reinforce each other. On the one hand, ROS production is dependent on key proteins of necroptosis, and it has been observed that TNF-induced ROS is suppressed after lowering the level of RIPK1/RIP3 proteins (129), on the other hand, oxidative stress induces necroptosis when sufficient amounts of ROS are present in the cell (130). ROS can activate early growth response gene 1 (Egr-1), which further activates RIPK1 and RIPK3, and initiating the necroptotic pathway. Mitochondria-targeted antioxidants can inhibit the production of mitochondrial ROS and reduce necroptosis (131).

Necroptosis has been linked to immune cell infiltration and has been demonstrated to take place throughout the pathogenesis of COPD (132). Necroptosis mediates the generation of airway mucus and stimulates inflammatory responses at the cellular level (133, 134). Blocking the necroptosis pathway can decrease inflammatory cell infiltration into the airway and lower airway wall thickness (135–137). Necroptosis exacerbates the inflammatory reactions that CS causes in macrophages, and in bone marrow-derived macrophages (BMDM), inhibiting necroptosis reduces the transcriptional levels of inflammatory cytokines that CS induces. The transcription factor NF-κB, which controls the expression of inflammatory genes, is typically made up of a P50/P65 heterodimer, in which P65 regulates NF-κB’s transcriptional activation. Necroptosis controls P65’s phosphorylation state, which controls the transcription of inflammatory genes (138). Through kinase activity, RIPK1 influences cell viability and death. Its activation can result in a highly inflammatory lytic form of cell death or apoptosis dependent on RIPK1 kinase, and blocking RIPK1 kinase can lessen the chance of airway remodeling (139).

## 8 Exosomes regulate airway remodeling by delivering bioactive substances

Extracellular vesicles (EVs) can be produced by almost all types of cells and are released when cells are activated, damaged, or apoptotic. Due to the complexity of EV origin, vesicle size is the most widely used parameter for distinguishing EV types. EV can be divided into three types according to particle size, the smallest of which is called exosome (140). Exosomes mediate cell-to-cell communication by delivering a variety of substances, including proteins, DNA, and RNA, to targeted cells (141, 142). Exosomes are involved in the development of COPD and airway remodeling (143). Oxidative stress is closely related to the production and function of exosomes. Oxidative stress can affect exosome production and release through multiple pathways, including regulation of lysosomal function and extracellular glutathione levels. It has been suggested that low levels of oxidative stress can promote the release of exosomes, while high levels of oxidative stress can inhibit their release. Oxidative stress can also affect the levels of proteins, lipids, and nucleic acids in exosomes (144). EV isolated from airway epithelial cells is also thought to induce fibroblast differentiation and increase the expression of type 1 collagen and α-SMA. CS stimulates airway epithelial cells to release exosomes, which transport microRNA-21(miR-21) to target cells. MiR-21 regulates the von Hippel-lindau protein (PVHL)/Hypoxia-inducible factors 1 (Hif-1) pathway, and promote fibroblast differentiation and EMT (143). When epithelial cells are exposed to CSE, EV-associated miR-422a is reduced, which causes fibroblasts to produce more type 1 collagen and α-SMA (145). Epithelial cell-derived exosomes also modulate inflammatory responses. The expression of miR-221 and miR-320 in exosomes from epithelial cell stimulates the secretion of pro-inflammatory cytokines, thereby activating AM, which in turn promotes inflammation (146). Immune cells present in the airways can release large amounts of EVs. EVs generated from granulocytes induced by bacterial formylated peptides carry more neutrophil elastase (NE). EV-bound NE cleaves type 1 collagen, causing alveolar expansion and higher airway resistance (147). T lymphocyte derived EVs from patients with COPD can stimulate bronchial epithelial cells to release pro-inflammatory factors and inhibit the synthesis of anti-inflammatory factors, causing inflammatory airway damage (148).

Extracellular vesicle may be a potential tool for the treatment of COPD by its substance-carrying capacity. As mentioned before, MSCs has been suggested to improve lung function in animal models with COPD. For MSCs are pluripotent stem cells that are capable of self-renewal and pluripotent differentiation into osteoblasts, adipocytes and many other cells (149). The MSCs can exploit its therapeutic advantage by releasing EVs. As with bone marrow (BM)-MSC-EVs, the therapeutic effect of the intraperitoneal combination of BM-MSCs and BM-MSC-Exosomes was explored in a study of CS-induced mitochondrial dysfunction in mice with COPD. The combination therapy alleviated COPD through anti-inflammatory and targeting mitochondrial genes, reducing the recruitment of neutrophils, CD4^+^ lymphocytes and macrophages in the bronchoalveolar lavage. Compared with BM-MSCs alone or their exosomes, the combined use of BM-MSCs and exosomes has a protective effect on the development of COPD (150). Placental Mesenchymal stem cell (PL-MSC)-derived products reduce inflammatory cytokine levels, reduce pulmonary macrophage and neutrophil infiltration, and enhance lung function (151). In a variety of fibrotic diseases, including pulmonary fibrosis, MSC exosomes have been shown to reduce fibroblast differentiation and reduce fibrosis (152).

## 9 Various herbal medicines may improve airway remodeling through antioxidants

The extract obtained from the whole herb of Polygonum aviculare L exerts anti-inflammatory activity. The ethyl acetate fraction of Polygonum aviculare L. inhibits the contraction of human ASM in mice and humans, and this inhibition is achieved by inhibiting calcium-permeable ion channels. In addition, Polygonum aviculare L. extract contains quercetin, which is also known to inhibit ASM contraction (153). Quercetin is a natural polyphenolic compound with a variety of pharmacological activities such as anti-inflammatory, antioxidant, and antitumor. It has been found that quercetin can enhance antioxidant defense by scavenging free radicals, inhibiting lipid peroxidation, and activating the Nrf2 pathway (154).

Epigallocatechin gallate (EGCG), the most abundant catechin analog in green tea, is a powerful scavenger of oxygen free radicals. It protects cells and DNA from damage. EGCG prevents CSE-induced oxidative stress, reduces ROS production and accumulation in airway epithelial cells, and inhibits the activation of NF-κB and the downstream expression of proinflammatory-type mediators (155).

Resveratrol inhibits pro-inflammatory cytokines and chemokines, and holds promise for treating COPD by reducing oxidative stress, inhibiting ROS, and suppressing inflammatory responses. Sirtuin 1 (SIRT1) and p38 MAPK are considered therapeutic targets for resveratrol (156, 157). In human umbilical vein endothelial cells (HUVECs), resveratrol attenuated CSE-induced endothelial cell apoptosis by inducing autophagy in a Notch1-dependent manner. In human airway epithelial cells, resveratrol attenuated CSE-induced cellular senescence by regulating the miR-34a/SIRT1/NF-κB pathway, suggesting a protective effect on vascular endothelial cells (158, 159).

Polyphenols are a class of natural phenolic compounds widely found in nature, with remarkable biological activities such as antioxidant, anti-inflammatory and anti-tumor. Polyphenols can exert a variety of functions by regulating the NF-κB and MAPK inflammatory pathways, the Nrf2 oxidative stress pathway, and the SIRT1/PGC-1α pathway, thus inhibiting airway inflammation, alleviating oxidative stress, reducing airway mucus hypersecretion, and restoring the balance between proteases and antiproteases (160). Curcumin, a natural polyphenol compound isolated from traditional Chinese medicine such as turmeric, was found to effectively attenuate airway inflammation and airway remodeling in COPD mice and inhibit the proliferation of human bronchial epithelial cells, which may be related to its inhibition of IκBα degradation and Cyclooxygenase-2 (COX-2) expression, suggesting that curcumin may reduce COPD airway remodeling through inhibition of the NF-κB signaling pathway and COX-2 expression. Curcumin can also activate the Nrf2 signaling pathway, which enhances the antioxidant defense of cells (161, 162).

Acute exacerbations are often induced by viral infections (such as influenza) in COPD patients, and the antiviral and anti-inflammatory effects of ZE339 may help to alleviate exacerbations. Ze339 is a CO2-extract from Petasites hybridus. It mainly contains compounds called petasins. ZE339 can not only inhibit viral replication, but also exert anti-inflammatory effects by inhibiting the signal transducer and activator of transcription (STAT) pathway and regulating cytokines. ZE339 may inhibit airway remodeling in COPD patients by inhibiting inflammatory response and cytokine release (163, 164).

## 10 Conclusion

One crucial aspect of COPD is airway remodeling, which controls the emergence of airflow restriction. The primary characteristics of airway remodeling include epithelial abnormalities, epithelial-mesenchymal transition, fibroblast differentiation and proliferation, and airway smooth muscle hypertrophy and proliferation. Multiple pathophysiological mechanisms are involved in airway remodeling, with oxidative stress being one of the most prominent. Air pollution and cigarettes may promote oxidative stress, leading to ASM proliferation, increased EMT, and influence ECM secretion. Inflammatory cell imbalance and dysfunction in the airways are also linked to elevated oxidative stress. Different oxidation levels influence the generation and release of exosomes that play a role in developing COPD and airway remodeling. In conclusion, better research is needed to understand the link between oxidative stress and airway remodeling. Conventional therapies for COPD consist of bronchodilators, glucocorticoids, antibiotics, and expectorants; nevertheless, they cannot limit airway remodeling successfully. Herbal treatment may improve COPD airway remodeling through antioxidants; further study of Chinese medicine is promising.
